# Women with PCOS who undergo IVF: a comprehensive review of therapeutic strategies for successful outcomes

**DOI:** 10.1186/s12958-023-01120-7

**Published:** 2023-08-01

**Authors:** Alexander M. Kotlyar, David B. Seifer

**Affiliations:** 1 Genesis Fertility/Maimonides Medical Center, Brooklyn, NY USA; 2 Section of Reproductive Endocrinology and Infertility, Department of Obstetrics, Gynecology, and Reproductive Sciences, Yale School of Medicine, Yale University, New Haven, CT USA; 3 Downstate Medical Center School of Medicine, State University of New York, Brooklyn, NY USA

**Keywords:** PCOS, In-vitro fertilization, Ovarian hyperstimulation, OHSS, AMH

## Abstract

Polycystic ovarian syndrome (PCOS) is a widespread syndrome that poses unique challenges and constraints to the field of assisted reproductive technology. This condition is the most common cause of anovulation among infertile couples. Debate exists over the best therapeutic course of action when patients with PCOS proceed to IVF. In this review, we evaluate the best-performing and safest methods of IVF preparation, ovarian stimulation, trigger method for maturation of stimulated egg growth, and planning for embryo transfer. Pre-IVF considerations include being aware of individual AMH and vitamin D levels as well as BMI prior to selecting an ovarian stimulation protocol. Numerous supplements such as myo-inositol complement the benefits of lifestyle change and may enhance IVF performance including oocyte yield and pregnancy rate. Concerning stimulation protocols, antagonist cycles with the judicious use of GnRH agonist trigger, pre-treatment with metformin and vitamin D repletion may help mitigate the accompanied risk of ovarian hyperstimulation syndrome (OHSS). Following ovarian stimulation, PCOS patients typically undergo programmed frozen embryo transfer (FET) cycles which are more conducive for women with irregular cycles, but likely carry a higher risk of hypertensive disorders of pregnancy. However, newer stimulated FET protocols using Letrozole may offer improved outcomes. Overall, patients with PCOS require careful individual tailoring of their IVF cycle to achieve optimal results.

## Background

Polycystic ovarian syndrome (PCOS) is the most common cause of anovulation, affecting up to 80% of women with this condition [1]. 9–18% of reproductive-age women have PCOS to some degree [2]. This diagnosis is based upon clinical and/or biochemical assessments of hyperandrogenism combined with ultrasound assessment for polycystic-appearing ovaries and/or oligomenorrhea as outlined by Rotterdam criteria. However, clinical features that may accompany this diagnosis can include obesity and other facets of the metabolic syndrome, mood disorders such as anxiety and depression and often infertility [3]. Women with PCOS are also noted to have higher serum anti-Mullerian hormone (AMH) levels with granulosa cells having up a 75-fold increased production of AMH [4]. Serum levels are 18-fold higher in anovulatory PCOS women than their ovulatory counterparts [5]. Given the known negative regulatory effect of AMH on FSH signaling and the inhibition of follicle development caused by AMH, elevated AMH is one mechanism which precludes cyclic follicular development in women with PCOS [6]. Numerous metabolic aberrations including the elevation in LH and elevated serum androgen levels also contribute to the increased AMH level [6–8]. These mechanisms, in addition to the effects of insulin resistance and hyperandrogenism, contribute to the anovulation and infertility seen in PCOS. Even when ovulation is present, PCOS patients may be at higher risk for infertility [9]. This may be due to oocyte quality issues and/or reduced endometrial quality [10, 11]. In addition, patients with PCOS who do ovulate and become pregnant, tend to experience higher rates of gestational diabetes, pre-eclampsia, and premature birth [12].

However, when pregnancy cannot be easily achieved, numerous treatments are available for infertility in the setting of PCOS. These include ovulation induction, with letrozole being the first-line agent with or without intra-uterine insemination, gonadotropin-IUI, laparoscopic ovarian drilling, and in-vitro fertilization (IVF) [1]. In a randomized-controlled trial (RCT) by Legro et al., patients treated with Letrozole exhibited both a significantly higher pregnancy rate (PR) and cumulative live birth rate (LBR) compared to treatment those treated with Clomiphene (PR: 41% vs. 27%, LBR: 28% vs. 19%, resp.) [13]. On average, women with PCOS required 90 days or approximately 3 cycles of Letrozole therapy to achieve a pregnancy [13].

Whether a patient will continue with further ovarian stimulation cycles or proceed with IVF depends on the combination of patient desire, value system (i.e. patience, risk tolerance, expense), age, and ovarian reserve. Currently, no cost benefit analysis has been done to clarify the most time- and cost-effective pathway once three or more cycles of Letrozole- or Gonadotropin-IUI have been completed. If the patient chooses to proceed with IVF, then numerous steps should be taken to optimize outcomes. Firstly, all patient pre-existing medical issues should be addressed prior to IVF to achieve the best and safest outcome. Secondly, one must assess how to achieve ovarian stimulation and triggering to maximize oocyte yield while minimizing the risk of ovarian hyperstimulation syndrome (OHSS). Thirdly, is the need to address how to best prepare the endometrium for implantation/pregnancy and mitigate pregnancy risks that may be encountered in this at risk population. In the following sections, we will address each of these important considerations.

## Methods

Our search utilized the following databases: Pubmed, The Cochrane Library, and Ovid-Medline. The phrases utilized for the search were adapted for each database and included “PCOS AND in-vitro fertilization”, “PCOS AND IVF”, “PCOS AND weight loss AND IVF”, “PCOS AND exercise AND IVF”, “PCOS AND myo-inositol”, “PCOS AND metformin”, “PCOS AND insulin sensitizer”, “PCOS AND Vitamin D”, “PCOS AND IVF stimulation”, “PCOS and trigger”, “Ovarian hyperstimulation AND PCOS”, “PCOS AND GnRH agonist”, “PCOS AND frozen embryo transfer”, “PCOS AND obstetric outcomes”, “PCOS AND perinatal outcomes”. Our search period spanned from 1946 to 2022. 6943 articles in total were found. Each of these articles was then evaluated based upon title and/or abstract for relevance (AK). Studies not published in English were excluded. 2604 duplicates were removed. Of the remaining studies, 59 of these articles were included within this review. The references of each cited source were assessed to so as not to exclude any other sources relevant to this review.

The primary focus was to evaluate the most recent literature on the role of IVF in patients with PCOS and on how to optimally prepare such patients for IVF, embryo transfer and the risks that may be anticipated in pregnancy. Articles were chosen for inclusion if they were: (1) retrospective or prospective studies or meta-analyses involving women with PCOS of reproductive age women and involved IVF and/or embryo transfer, (2) Systematic reviews or (3) incorporated in-vitro or in-vivo animal or cell culture studies in which signs of PCOS or ovarian hyperstimulation syndrome were recapitulated. Excluded studies (1) were case reports, case series, abstracts, expert opinion articles, (2) did not involve patients with PCOS or (3) did not involve patients undergoing IVF and instead undergoing procedures such as intra-uterine insemination (IUI) or in-vitro maturation (IVM).

## IVF preparation

Even before IVF has been planned, a proper assessment should be taken to optimize IVF cycle outcomes in a patient with PCOS. These include lifestyle modification, cycle priming and various adjuvants i.e. supplements/medications that could address aspects of PCOS pathophysiology. Elevated age-specific AMH levels, decreased vitamin D levels and elevated BMI (≥ 30) provide insight into choosing metformin pretreatment and/or vitamin D supplementation. During IVF, choosing the optimal method of ovarian stimulation and triggering method are essential in lowering chances of OHSS and maximize egg yield.

### a. Metabolic Assessment and Lifestyle adjustment:

PCOS is a condition with strong associations with metabolic disturbances such as those seen metabolic syndrome. Metabolic syndrome is a constellation of physical and metabolic abnormalities which involves signs of insulin resistance, excess weight, hypertension, and hyperlipidemia. Currently the Adult Treatment Panel III  (ATPIII) guidelines are used to establish the diagnosis; however, even if these criteria is not met, many PCOS patients may exhibit some aspect of metabolic syndrome [1, 14]. One of the chief abnormalities is insulin resistance, the gold standard to assess this is the euglycemic insulin clamp test; however, this test is generally limited to research scenarios [1]. A more common assessment relies on a glucose tolerance test (OGTT) using a 75g glucose load. Given the available data and relative cost, the OGTT is considered the standard for assessing impaired glucose tolerance in at-risk PCOS patients [15]. Approximately 35% of PCOS patients will have some form of insulin resistance and 10% will meet criteria for diabetes mellitus [16]. Other metabolic syndrome abnormalities often seen with PCOS include hyperlipidemia, especially in obese patients. Hence all obese women should undergo a fasting lipid profile [17]. Once a PCOS patient has been assessed for the abnormalities, interventional steps can be started.

Our current understanding of the pathophysiology of PCOS highlights the need for lifestyle modification as a path to better clinical outcomes. Weight and its relationship to insulin resistance and circulating free fatty acids are key drivers of PCOS pathophysiology. Hence, mitigating excess weight is considered beneficial for women with PCOS. Numerous studies have highlighted the benefit of weight loss as a method to improve reproductive outcomes in this population [1]. Even a 5–10% weight loss in PCOS patients can lead to resumption of normal ovulation [1]. Gao et al assessed the performance of BMI, cholesterol and basal FSH on the IVF outcomes in patients with PCOS and their predictive model showed an AUC of 0.708 for live birth rate, suggesting some predictive role for metabolic parameters [18]. Weight can also impact fertilization outcomes with overweight PCOS patients having a 69% lower PR per cycle start and a 71% lower LBR compared to lean PCOS patients [19]. Physical activity has also been shown to be beneficial; in a pooled analysis, Mena et al found a higher PR and LBR in PCOS patients undergoing consistent physical activity compared to those undergoing dietary or pharmaceutical therapy alone [20].

Once a patient’s lifestyle has been optimized as far as weight loss and physical activity, the question then arises as to how to best prepare for the actual IVF cycle. Pre-treatment with combined oral contraceptives (COCs) or estradiol to help synchronize the nascent follicles is one common approach. One nested cohort study from 2017 indicated that women with PCOS who started IVF following a spontaneous menses had a higher pregnancy rate (PR) and live birth rate (LBR) compared to those that were on COCs prior to their IVF cycle [21]. Luteal phase estradiol supplementation has not been as well studied in this population. However, early supplementation of estradiol in at least one study showed greater numbers of retrieval metaphase II (MII) oocytes in PCOS patients [22]. Considering these findings, while it would be best to avoid COCs prior to an IVF cycle, COCs may still need to be used to ensure optimal cycle timing for various logistical purposes including patient convenience.

### b. Adjuvant agents

#### 1. Myoinositol

As a natural insulin sensitizer, myo-inositol has been studied extensively in PCOS patients [23, 24]. Papaleo et al in their small RCT showed that patients treated with myo-inositol and 2g per day of folic acid needed less gonadotropin during stimulation and had fewer number of immature oocytes [25]. A subsequent meta-analysis incorporating, 8 studies on myo-inositol showed a consistent decrease in gonadotropin dose and duration of ovarian stimulation in PCOS patients undergoing IVF [26]. Zheng et al completed a meta-analysis of seven trials, four of which involved patients with PCOS which confirmed higher clinical PRs with lower gonadotropin amounts in those patients undergoing IVF and pre-treated with myo-inositol [27].

Melatonin has also been studied in combination with myo-inositol. This molecule is an essential regulator of not just circadian rhythm, but also is key in obtaining adequate oocyte quality. This is chiefly due to its activity as a free-radical scavenger which can help mitigate the effects of oxidative stress, the main contributor to ovarian aging. Hence, melatonin is considered to be a factor which can enhance proper oogenesis [28]. Based upon work showing disruptions in melatonin signaling in PCOS patients, a subsequent prospective study looked at the addition of melatonin in PCOS patients undergoing IVF/ICSI and found a greater number of mature oocytes with a trend towards higher implantation and clinical PRs [29, 30]. The same group assessed the effect of myo-inositol with melatonin on PCOS patients who did not conceive in previous IVF cycles. In 46 patients undergoing treatment, 13 went on to conceive. While encouraging, this study was severely limited by the lack of a control group. One subsequent controlled prospective trial and one RCT confirmed that oocyte and embryo quality improved following treatment with myo-inositol, folic acid, and melatonin [31, 32].

Concerning folic acid, its effect in combination with myo-inositol was reinforced by the work of Wdowiak et al which showed an improvement embryo/blastcocyst formation and PR in patients with PCOS supplemented with 4g of myo-inositol and 400 mcg of folic acid per day compared to PCOS patients only given folic acid [33]. It is key to note that approximately 38% of patients may be resistant to myo-inositol, but studies in which alpha-lactalbulmin was supplemented noted reduction in this resistance. However, these studies were not randomized trials and did not involve patients undergoing IVF thus severely limiting general applicability [34–36]. Overall myo-inositol especially when combined with folic acid and melatonin may be a promising adjuvant for any patient with PCOS planning IVF.

#### 2. Insulin sensitizers

Insulin resistance has previously been shown to have a negative correlation with ovarian sensitivity [37]. Metformin has been the most commonly-studied insulin sensitizer in PCOS patients. Tang randomized patients to 850mg of Metformin versus placebo from the day of ovarian suppression to the day of retrieval and noted a greater clinical pregnancy rate after 12 weeks and a lower rate of OHSS in the Metformin group [38]. A more recent small RCT with 102 overweight and obese PCOS patients randomized to 1000mg of Metformin at time of ovarian stimulation versus placebo showed lower oocyte yield, but similar LBR in the Metformin group [39]. In contrast, Jacob et al in their RCT noted lower clinical PR per cycle started and lower LBR, but a lower risk of OHSS [40]. Tso et al later completed a systematic review and meta-analysis in women on the effect of Metformin before or during IVF/ICSI cycles and found no difference LBRs, but OHSS risk overall was observed to be lower [41]. Other insulin-sensitizing agents have also been studied. In a small RCT, Salamun et al found that Liraglutide, a GLP-1 receptor agonist, in combination with Metformin led to higher cumulative PR over 12 months in women with PCOS undergoing IVF. It is anticipated that GLP-1 receptor agonists may be more commonly utilized in the future as increased clinical experience accumulates and both short and long-term neonatal and infant studies are published. Of note, both the Metformin only group and the Metformin and Liraglutide group experienced a similar weight loss of an of average 7-7.5kg [42].

Insulin sensitizers remain an attractive adjuvant in treating PCOS patients planning IVF. While OHSS risk may be mitigated and some limited weight loss may be achieved, it is unclear if these agents could improve oocyte yield and resultant PR and LBR.

#### 3. Vitamin D

Lower than normal Vitamin D levels have been linked to abnormal metabolic outcomes in PCOS patients [43, 44]. Numerous groups have assessed the effect of Vitamin D on outcomes in such women undergoing IVF. Abadia et al performed a cross-sectional study looking at Vitamin D levels in women undergoing ART and noted a positive correlation between vitamin D levels and oocyte fertilization rates. However, no correlation was noted with pregnancy outcomes [45]. This reinforced a prior Iranian study looking at follicular and serum levels of vitamin D. Unfortunately, both the pregnant and non-pregnant patients had Vitamin D levels below 10ng/mL which likely biased their results [46]. However, a subsequent prospective cohort study looking at Vitamin D levels and pregnancy rates following frozen embryo transfer did not show any correlation, especially after an adjusted analysis [47]. Chu et all then did a much larger prospective cohort study and observed higher LBR for patients that were Vitamin D replete (37.7%) compared to deficient (23.2%) and insufficient (27.0%) patients [48], yet this barely achieved statistical significance (p = 0.04).

Additional parameters notable for women with PCOS undergoing IVF is a risk of OHSS which is a believed to be driven by elevated VEGF levels. A small RCT noted lower VEGF levels in vitamin D deficient PCOS patients treated with aggressive Vitamin D supplementation [49]. The same group also noted improved levels of numerous other metabolic and inflammatory signals [50, 51].

Overall, while abnormally low vitamin D levels have been associated with PCOS, the role of vitamin D measurement and supplementation in PCOS patients undergoing IVF is still unclear. Further high quality RCTs which include both reproductive outcomes and OHSS rates are crucial to better understanding the role of this nutrient.

## Ovarian stimulation

### a. Stimulation method

Once an IVF cycle is planned, the actual ovulation induction protocol can have a significant impact upon outcomes. Thus, the question arises of whether a standard-long GnRH-agonist protocol versus a GnRH antagonist protocol is preferable. Lanias et al conducted an RCT with 220 patients comparing the two protocols and found lower OHSS rates, lower gonadotropin doses and lower stimulation duration in patient that underwent a GnRH-antgonist protocol [52]. A later phase IV, open-label RCT involving 1050 first IVF/ICSI cycles showed a lower risk of OHSS and its complications when a GnRH-antagonist protocol compared to using a standard-long GnRH-agonist protocol [53]. While not solely focused on PCOS patients, this study reinforced the concept that GnRH-antagonist cycles can lead to reduced OHSS risk. A meta-analysis from 2022 looking at 10 RCTs which confirmed the lower risk of OHSS using a GnRH-antagonist protocol, but a lower retrieved oocyte number. Despite the lower oocyte yield, there was no difference in PR and LBR and miscarriage rate compared to patients that underwent a standard-long GnRH-agonist protocol [54]. A subsequent meta-analysis of 50 RCTs comparing antagonist versus the standard long-agonist protocol showed lower ongoing pregnancy rates (RR 0.89, 95% CI 0.82–0.96). Yet, OHSS rates were substantially lower in PCOS patients that were treated with an antagonist protocol (RR 0.53, 95% CI 0.30–0.95). It must be noted that a contributor to the lower OHSS risk in patients in the GnRH-antagonist protocol was the use of a GnRH agonist trigger. In short, GnRH antagonist protocols seem to offer the best combination of cycle flexibility and OHSS risk minimization for PCOS patients.

An alternative protocol involves suppression of LH using progestins at the time of ovarian stimulation. A prospective cohort study by Xiao et al compared a progestin-suppression (termed progestin-primed) protocol compared to a flexible GnRH antagonist approach and found that progestin-primed patients had similar pregnancy rates and lower OHSS rates, but the dose and duration of gonadotropin treatment was greater [55]. In a retrospective study with 333 women with PCOS, progestin suppression in lieu of a GnRH antagonist led to similar PR and LBR with no increased risk of a premature LH compared to a GnRH antagonist protocol [56]. These aforementioned results are encouraging, but additional multicenter RCTs are necessary to best further elucidate the effectiveness of this progestin-based protocol in PCOS patients.

Minimal stimulation IVF has also been studied in the PCOS population. This protocol is meant to grow a cohort of follicles with a maximum dose of 150 IU of FSH [57]. A retrospective study of 235 cycles from Germany comparing IVF outcomes in patients with and without PCOS noted no difference in clinical PR and OHSS rates [58]. A recent meta-analysis of 31 RCTs indicated that the use of minimal-stimulation IVF exhibited similar live-birth rates compared to conventional dose IVF in high responders such as PCOS patients [59]. Hence, minimal stimulation IVF presents a tantalizing option for PCOS patients especially if OHSS risk is excessive and/or cost must be minimized.

### b. Trigger method

Once a patient has undergone ovarian stimulation and has achieved follicles of sufficient size likely to obtain mature oocytes, the next step is to choose the most effective trigger injection. Recombinant hCG is the established standard for most protocols [3]. However, PCOS patients tend to have a higher risk for OHSS and hCG is not necessarily the best option in this case [3]. Engmann did an RCT of sixty-six patients with PCOS or a history of high response during IVF and showed that triggering with a GnRH agonist led to lower rates of OHSS compared to using hCG [60]. A subsequent meta-analysis incorporated this and 16 other RCTs and determined that the risk of OHSS was substantially reduced using a GnRH trigger compared to an hCG trigger (OR 0.15, 95% confidence interval (CI) 0.05 to 0.47). However, without an adjustment of added luteal support, patients that received the GnRH agonist trigger had a somewhat lower LBR and higher miscarriage rate [61]. Numerous prospective and retrospective studies have indicated that luteal support, in the form of addition hCG or LH can improve implantation rates and LBR in patients receiving an agonist trigger to that of patients receiving an hCG trigger [62–64].

In addition to a using GnRH agonist as a trigger to minimize OHSS, other adjuvants around the time of the trigger injection have been evaluated. The most well-studied is the use of dopamine agonists, the most commonly used of which is cabergoline. The use of dopamine agonists is based upon animal models of OHSS and evidence of low-dopamine tone in PCOS patients leading to dysregulated VEGF signaling (the key factor behind the pathogenesis of OHSS) [65, 66]. Cabergoline inhibits vascular endothelial growth factor receptor 2 (VEGFR-2) phosphorylation and signaling thereby preventing VEGF’s action on its receptor and thus mitigating OHSS. An RCT compared cabergoline at the time of trigger and placebo and treatment with cabergoline decreased the moderate OHSS risk to 20% compared to 43.8% with placebo. In addition, patients experienced smaller increases in their hemoglobin and the accumulation of ascites [67]. Concerning the timing of cabergoline administration, a recent retrospective study compared dosing at time of GnRH agonist versus day of retrieval with the former group exhibiting less mild-to-moderate OHSS [68]. This approach of providing cabergoline at time of trigger instead at time of retrieval makes inherent sense given the need to prevent any early rise in VEGF levels. Additional studies have assessed other agents to supplement cabergoline such as the use of luteal GnRH antagonists which is effective while the use of albumin as an intravascular volume expander is less effective [69, 70]. Given the above literature, the use of cabergoline is recommended in patients experiencing OHSS according to the American Society for Reproductive Medicine (ASRM). However, the European Society for Human Reproduction and Embryology (ESHRE) does not recommend using cabergoline if a GnRH agonist trigger has already been used.

### c. Fresh transfer versus frozen transfer

Once a PCOS patient has undergone successful oocyte retrieval and has obtained embryos, several decision points arise concerning the fresh versus frozen-thaw approach to embryo transfer. Given the altered hormonal milieu in PCOS and the typically higher estrogen levels in these patients, the question of performing a fresh transfer versus a freeze all strategy followed by a thaw embryo transfer has also been considered. Chen et al performed one of the largest RCTs to address this question and their data indicated that a freeze-all strategy for PCOS patients led to a higher LBR, lower miscarriage rate, and lower OHSS rate. It is of note that this study only performed cleavage-stage transfers [71]. A subsequent RCT using 212 high-responding patients (as PCOS patients tend to be) showed no difference in PR and LBR between the freeze-all and fresh transfer with hCG-support arms. Both cleavage-stage and blastocyst-stage embryos were transferred. However, the fresh transfer arm was the only one to exhibit moderate to severe OHSS at a rate of 8.6% compared to 0% [72]. Overall, a freeze-all strategy appears to yield better outcome as far as LBR and lower OHSS for patients with PCOS.

## Frozen thaw embryo transfer (FET)

If a freeze-all strategy has been adopted whether for OHSS-mitigation, desire for pre-implantation genetic testing or potentially other patient/provider preferences, the method and timing of subsequent frozen embryo transfer is a crucial question. Given that oligovulation is present in a majority of PCOS patients, programmed (hormone replacement) FET cycle protocols have been extensively used and studied in these patients [1]. Man et al performed a retrospective cohort analysis on PCOS patients undergoing various endometrial preparation regimens prior to FET. These were natural cycle, ovarian stimulation, and hormone replacement. The pregnancy rates for each method were 72.3, 73.8, and 64.9% with LBRs being 62.4, 65.0, and 52.2%, respectively, with the later achieving statistical significance (p < 0.009) [73]. In the meta-analysis of Kollmann et al, a comparison of a human menopausal gonadotropin-simulated FET protocol versus a hormone replacement cycle using estradiol valerate showed no difference in LBR [74]. Despite the mixed data concerning the success rates of various FET protocols in PCOS patients, hormone replacement protocols provide the greatest degree of flexibility and predictablility in planning an embryo transfer.

Given this data on programmed FET protocols, one must ask if any role remains for natural cycle FET. The answer to this remains a resounding, ‘yes’. If a patient wishes to minimize exposure to exogenous hormones whether this be for personal preference versus a medical indication (e.g. history of estrogen/progesterone receptor-positive breast cancer), then a natural cycle FET could still be attempted. In addition, there is substantial data on using Letrozole to stimulate monofollicular growth. Zhang et al did a retrospective study of 2664 patients comparing a Letrozole-stimulated FET protocol compared to a hormone replacement protocol and found a greater LBR for the Letrozole-stimulated FET group. However, a subsequent meta-analysis analyzing outcomes from four retrospective cohort studies found no difference in PR or LBR for letrozole-stimulated cycles compared to programmed FET cycles [75]. Of note, letrozole-stimulated FET cycles have been associated with lower risk of hypertensive disorders of pregnancy compared to programmed FET cycles [76].

A note must be made here about endometrial receptivity. The altered hormonal milieu in PCOS patients with higher androgens and higher estrogen levels at the time of ovarian stimulation is believed to be deleterious to endometrial receptivity and embryo implantation [77] thus, further supporting the preference for frozen-thaw transfer in lieu of a fresh transfer.

## Obstetric and Perinatal Outcomes

Once a PCOS patient has achieved a pregnancy via IVF, the goal then becomes precluding adverse events during pregnancy. Numerous studies have shown an adverse effect of PCOS on general perinatal outcomes, especially when that patient is overweight or obese. These adverse effects include a higher risk of gestational diabetes (GDM), hypertensive disorders of pregnancy (HDP), pre-term birth (PTB) and macrosomia and seem to be independent of diagnostic criteria [78–80].

When examining the population of PCOS patients undergoing IVF, a more complex picture appears. Wan et al did a retrospective cohort study looking at 864 patients, of the 54 live births in the PCO group and 174 in the control group, they did not notice any difference in the rates of GDM, HDP, and intrauterine growth restriction (IUGR) [81]. Ectopic pregnancy rates were assessed by Wang et al and in their analysis they noted a higher risk of ectopic pregnancy following fresh embryo transfer 7.0% vs 2.4% adjusted odds ratio [aOR], 3.06; 95% confidence interval [CI], 1.34–6.96). This effect was absent when frozen transfers were compared between PCOS and non-PCOS patients thus further supporting a freeze-all strategy. Notably, for women with PCOS who underwent FET, pre-pregnancy weight overall did not lead to any differences in perinatal outcomes aside from an increased risk of cesarean delivery in patients who are overweight and obese [82]. Overall, the totally of data suggests that PCOS patients undergoing IVF are at higher risk for specific obstetric adverse events such as HDP and that excess weight can exacerbate overall risk.

## Conclusions

PCOS remains the most common cause of anovulation among women with infertility. When women with PCOS require IVF to treat their infertility, numerous beneficial interventions can be adopted that may maximize not only pregnancy rates but also the ability to achieve a live birth while minimizing the iatrogenic risk of OHSS. They include lifestyle modification which can aid in weight loss and potentially enhance IVF outcomes. In addition to lifestyle modification, numerous adjuvants especially myo-inositol (supplemented with melatonin and/or folic acid) can enhance oocyte quality and potentially IVF pregnancy rates. While the evidence concerning vitamin D supplementation is tantalizing, additional RCTs are necessary before its role can be understood in enhancing IVF success rates. Additional strategies which can improve outcomes in PCOS patients include, using GnRH antagonist protocols for IVF stimulation to minimize OHSS risk. OHSS risk can also be mitigated using vitamin D repletion, GnRH agonist triggers and dopamine agonists following oocyte pick-up. As embryos are obtained, deferring embryo transfer until a subsequent cycle by adopting a freeze-all strategy can further enhance outcomes by optimizing endometrial receptivity. Finally, while programmed FET protocol can overcome the lack of consistent ovulation, limited evidence indicates that using a Letrozole-stimulated FET protocol can offer similar pregnancy rates and potentially improve obstetrical outcomes.

Despite these advances, there is ever room for improvement and additional questions remain as to how IVF outcomes can be further enhanced in PCOS patients. These include methods to further assess and enhance endometrial receptivity as well as methods to limit obstetrical complications in those patients undergoing programmed FET. This will require additional diligent analysis of the biochemistry and pathophysiology of PCOS and a deeper understanding of the dynamics of embryo implantation.

## Data Availability

Not applicable, please ‘Methods’ section for article search strategy.

## Abbreviations

PCOS: Polycystic ovarian syndrome
PR: pregnancy rate
LBR: live birth rate
OHSS: ovarian hyperstimulation syndrome
COCs: combined oral contraceptives
VEGFR-2: vascular endothelial growth factor receptor 2
FET: Frozen thaw embryo transfer
GDM: gestational diabetes
HDP: hypertensive disorders of pregnancy
PTB: pre-term birth
IUGR: intrauterine growth restriction
